# Hydration Assessment Using the Bio-Impedance Analysis Method

**DOI:** 10.3390/s22176350

**Published:** 2022-08-24

**Authors:** Reem AlDisi, Qamar Bader, Amine Bermak

**Affiliations:** 1 College of Health and Life Science, Hamad Bin Khalifa University, Doha 34110, Qatar; 2 Department of Electrical and Computer Engineering, Faculty of Engineering and Applied Science, Queen’s University, Kingston, ON K7L 3N6, Canada; 3 College of Science and Engineering, Hamad Bin Khalifa University, Doha 34110, Qatar

**Keywords:** bio-impedance analysis, COMSOL Multiphysics, hydration assessment, inkjet printing, interdigitated electrode, wearable devices

## Abstract

Body hydration is considered one of the most important physiological parameters to measure and one of the most challenging. Current methods to assess hydration are invasive and require costly clinical settings. The bio-impedance analysis offers a noninvasive and inexpensive tool to assess hydration, and it can be designed to be used in wearable health devices. The use of wearable electronics in healthcare applications has received increased attention over the last decade. New, emerging medical devices feature continuous patient monitoring and data collection to provide suitable treatment and preventive actions. In this paper, a model of human skin is developed and simulated to be used as a guide to designing a dehydration monitoring system based on a bio-impedance analysis technique. The study investigates the effect of applying different frequencies on the dielectric parameters of the skin and the resulting measured impedance. Two different interdigitated electrode designs are presented, and a comparison of the measurements is presented. The rectangular IDE is printed and tested on subjects to validate the bio-impedance method and study the interpretation of its results. The proposed design offers a classification criterion that can be used to assess dehydration without the need for a complex mathematical model. Further clinical testing and data are needed to refine and finalize the criteria.

## 1. Introduction

Water is an essential component of the human body, and its balance is vital for many internal functions such as temperature regulation and metabolism [1,2]. On a daily basis, water is lost from the kidneys, lungs, and skin, and this loss must be compensated by water intake [3]. Mild levels of dehydration (1–2%) have been shown to affect body physiology [2]. Research has shown that dehydration causes reduced endurance, increased fatigue, and altered thermoregulatory capability. Research has also shown that cognitive functions such as concentration and alertness are affected by the status of hydration [4]. Hydration status can also be an indicator of several diseases that distort whole-body fluid and electrolyte balance [2]. Hence, assessing hydration status accurately and reliably is important in health monitoring.

Over the years, different techniques have been explored and used to evaluate hydration accurately. These techniques vary between being qualitative and quantitative, invasive and noninvasive measurements, and laboratory tests [3]. These methods include the dilution technique, blood tests, urine tests, and body mass difference measurement. While these methods can give results with accuracies of 1% to 2%, they require clinical settings, and the results are not immediate [2,3].

Recent research has focused on developing the use of bio-impedance analysis methods as a noninvasive, inexpensive, and safe tool to evaluate hydration status. Bio-electric impedance analysis (BIA) relies on the fact that body tissue can be modeled as an electrical circuit consisting of resistances and capacitance [5]. The relationship between the measured impedance and hydration level has been studied throughout the literature [6,7,8,9], and several equations have been empirically formulated to express the total body water as a function of impedance, height, weight, and sometimes age and sex [5]. The BIA method has the potential to be designed and implemented as a wearable device using different technical set-ups [10,11,12]. However, there is a need for manually tuning the device since some parameters such as height, weight, and age need to be input, and any device based on this scheme will require regular calibration, which is a challenge if the system is to be deployed on a large scale.

In this work, a study on the use of the bio-impedance analysis technique in measuring skin hydration is presented. A human skin model is simulated, taking into account the dielectric properties in the case of hydration and dehydration. Skin electrodes are used to apply an AC signal to measure skin impedance. The AC/DC module in COMSOL was used to study the effect of applying different frequencies on the dielectric parameters of the skin and the resulting measured impedance. The rectangular interdigitated electrode design was studied as the main and preferable design. The electrode was then fabricated, and the method was tested and validated by comparing the resulting parameters with the hydration measurement of the BodyStat Multiscan 5000 body fat analyzer and hydration monitor. The simulated work is proposed as a guide to designing a wearable dehydration monitoring system to assist subjects working in extreme and difficult conditions, such as construction workers.

The remainder of this article is organized into four sections: Section 2 will present the methodology used. Section 3 will show the results. Section 4 will present a discussion of all work performed, and Section 5 will conclude the paper.

## 2. Methodology

In this section, the methodology used to achieve the aims of this paper is presented. The methodology is divided into three main parts. The first part looks into the design aspects of the electrodes in the system. The second part focuses on the simulation model, which uses the skin model presented in [13] to study the designed electrodes. The third and final part explains the testing and validation procedure followed in this work.

### 2.1. Electrode Design

To design the electrodes, the fabrication process to be used and various parameters must be taken into consideration. Traditionally, wearable devices have been manufactured by adopting different techniques, including photolithography, vacuum deposition, and electrode plating process. However, these techniques are complicated, costly, and not environmentally friendly. Recently, the usage of inkjet printing in fabricating wearable electronics has been widely investigated in the literature. With its simple process, inkjet printing provides a highly efficient, cost-efficient, and eco-friendly fabrication means [14,15,16,17]. Ink printability, substrate properties, and pattern uniformity and resolution are critical factors in inkjet printing and must be carefully considered in the design process [15].

For wearable electronics, conductor-based inks are the most used, including metals, conducting polymers, and other organic materials, such as carbon-based materials. Due to their conductivity, metals are deployed in printing electronics, with gold (Au) being the most superior and most biocompatible metal. However, because of its relatively acceptable cost, the most employed metal is generally silver (Ag). Some other metals, such as copper (Cu) and aluminum (Al), while being economically a great solution, are easily oxidized and can introduce a challenge in the fabrication process. Carbon-based materials also present a challenge in fabrication, despite their high conductivity, chemical stability, and flexibility, due to their poor dispersion in solvents. Conducting polymers, such as polypyrrole (PPy), are easy to process, flexible, biocompatible, and low-priced, yet are less conductive compared to metals and carbon-based materials [15].

Droplet size, which is determined by nozzle diameter and waveform, plays a huge role in determining the resolution of the printer used in fabrication. Generally, inkjet printers can print up to 100-nm-thin layers with a moderate resolution of approximately 50 μm [18]. These considerations must be taken into account when designing the circuitry printed by inkjet technology.

**Figure 1:**
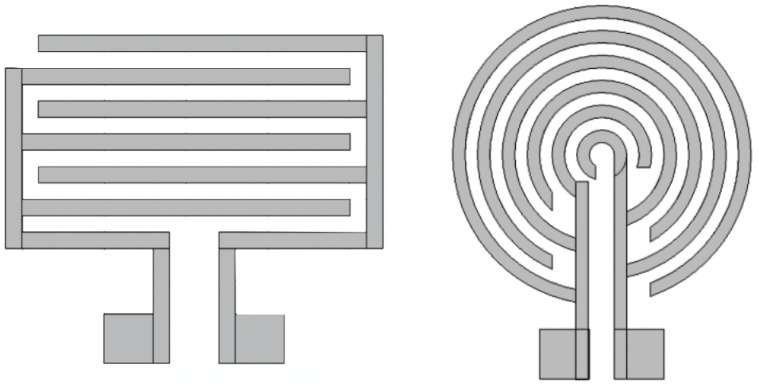
Electrode designs: rectangular IDE (**Left**), circular IDE (**Right**).

Two sensor structures have been investigated and designed. Both of which are interdigitated to maximize the effective area of the electrodes. IDEs have been widely used and favored, especially among chemical and biological sensors, because of their high sensitivity, ease of fabrication, and low cost. Figure 1 illustrates a typical design of a circular and rectangular IDE.

To execute a fair comparison, the geometry parameters of both designs have been unified to have the specifications in Table 1.

In both sensors, the two electrodes form a capacitor in which the first electrode is the exciting electrode, where an alternating current is injected with a frequency ranging from 100 Hz to 0.1 MHz, and the second electrode is the sensing electrode, where the output is measured. Between fingers, a fringing field is formed due to the electrodes being placed in the same plane, making the electric field penetrate the material under test. The fringing field enables the measurement of electrical impedance, which reflects the water content of the skin. When the skin is hydrated (i.e., it has more water molecules), it becomes more conductive and, therefore, less resistant to current flow and vice versa for the dehydrated skin.

### 2.2. Simulation

To model and simulate the designed electrode, the skin model in [13] was used. The skin model, which was designed to study and validate the BIA method, uses the dielectric parameters presented in [19,20,21] for the hydrated and dehydrated skin. The BIA method was validated by using the AC/DC module in COMSOL Multiphysics, which helps in providing a detailed analysis of the electrical properties of the simulated model. In [13], a simple model was first simulated by applying an alternating signal to one electrode with a frequency ranging from 100 Hz to 0.1 MHz, while the other electrode was set to the ground.

**Figure 2:**
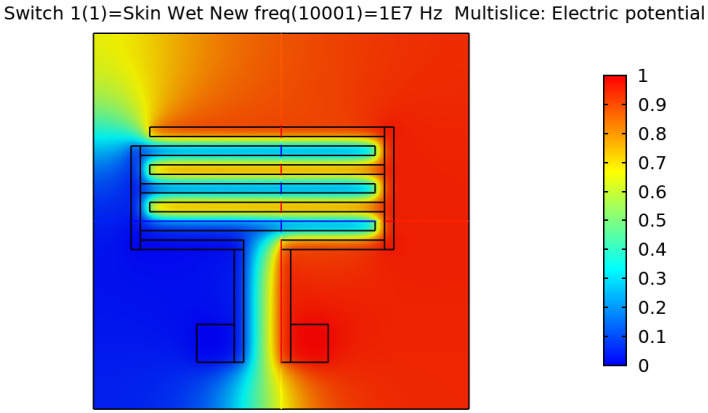
Electric distribution (V) in 3D mode for the rectangular IDE.

**Figure 3:**
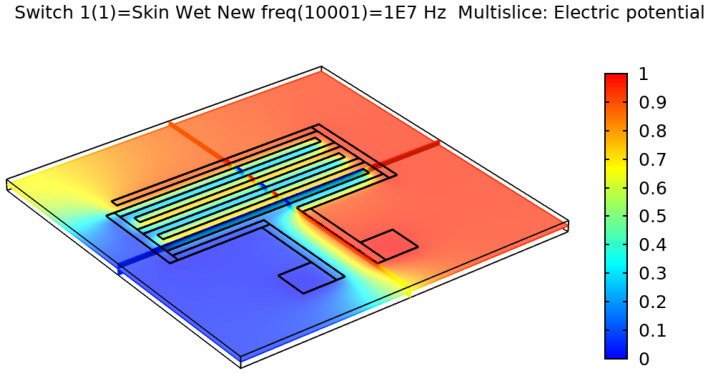
Electric distribution (V) in 3D mode for the rectangular IDE.

**Figure 4:**
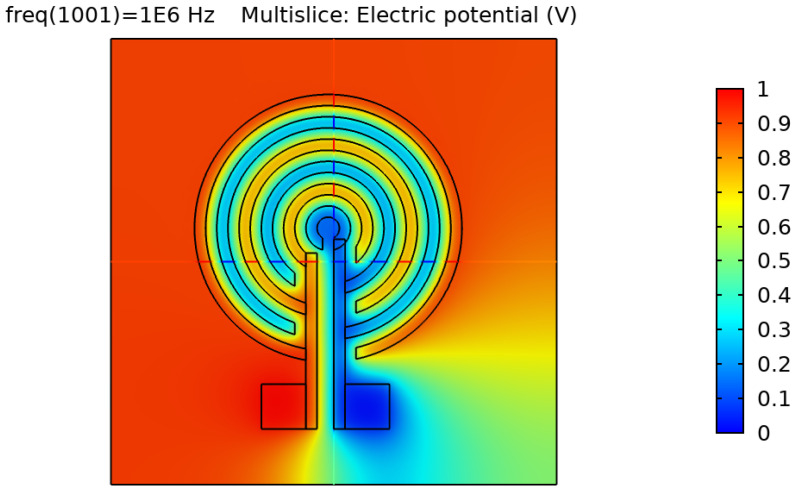
Electric distribution (V) in 3D mode for the circular IDE.

**Figure 5:**
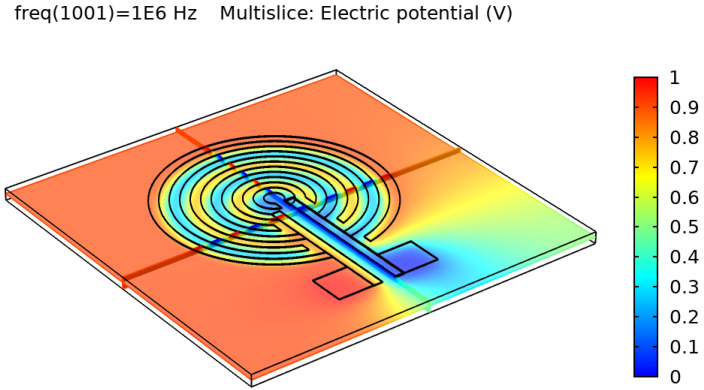
Electric distribution (V) in 3D mode for the circular IDE.

After validating the skin model, a 3D model of each electrode design was simulated and tested. Figure 2, Figure 3, Figure 4 and Figure 5 show the resulting electric distribution of the skin when the signal is applied to one electrode while setting the second electrode to the ground. As noticed, the distribution shows the changes of potential around the electrodes and between the fingers of the electrode.

**Figure 6:**
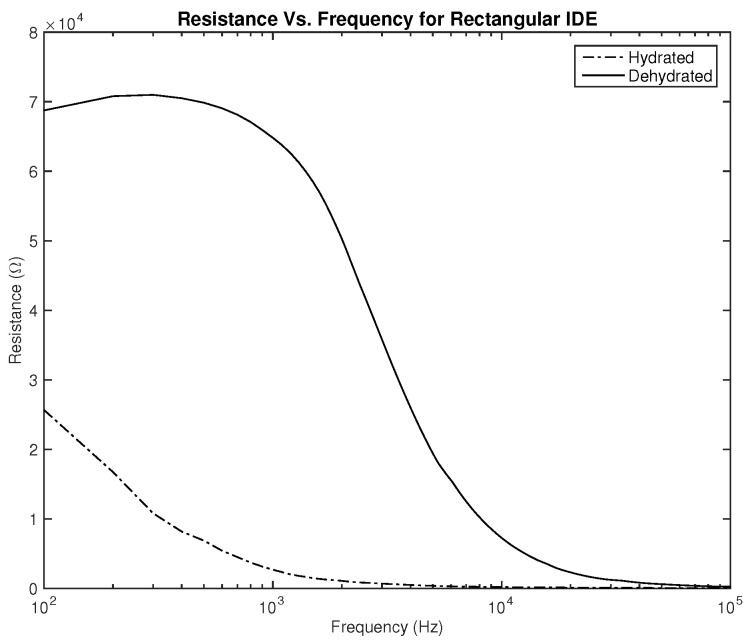
Simulation results—resistance (Ω) of hydrated and dehydrated skin using the rectangular IDE design.

**Figure 7:**
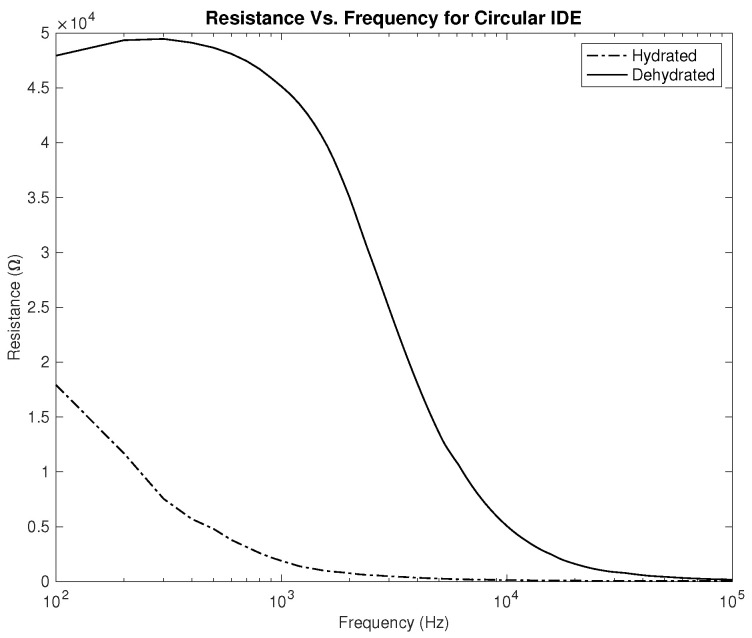
Simulation results—resistance (Ω) of hydrated and dehydrated skin using the circular IDE design.

**Figure 8:**
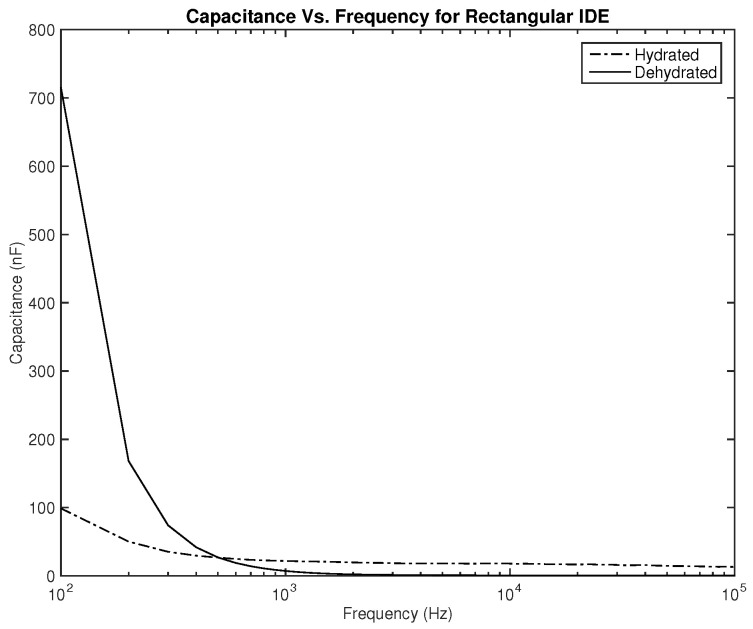
Simulation results—capacitance (nF) of hydrated and dehydrated skin using the rectangular IDE design.

**Figure 9:**
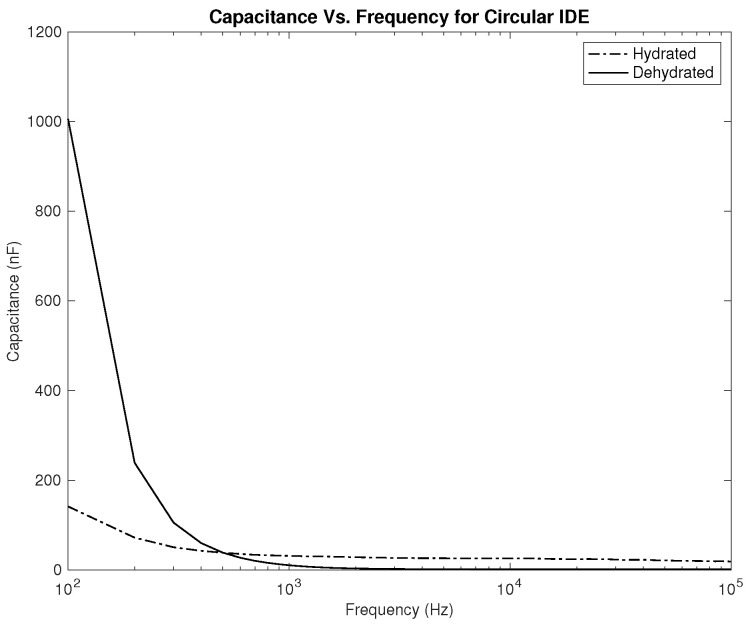
Simulation results—capacitance (nF) of hydrated and dehydrated skin using the circular IDE design.

**Figure 10:**
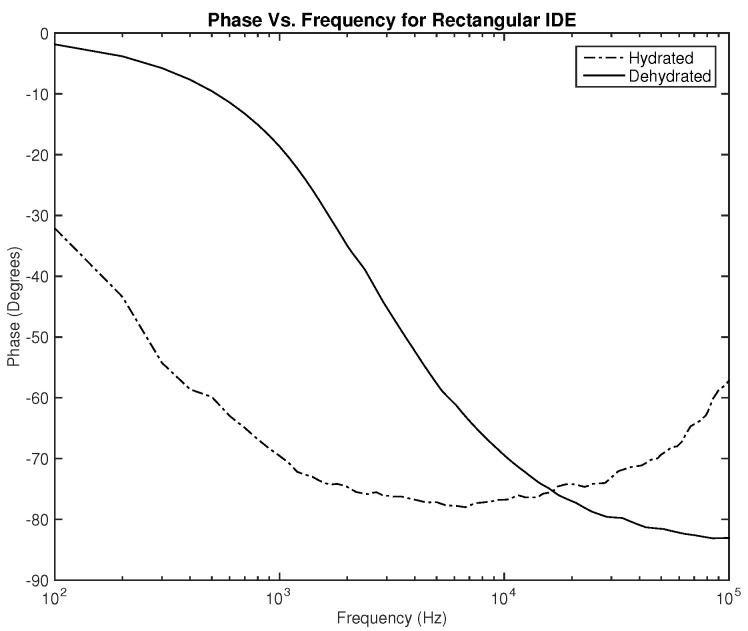
Simulation results—phase (Degree) of hydrated and dehydrated skin using the rectangular IDE design.

**Figure 11:**
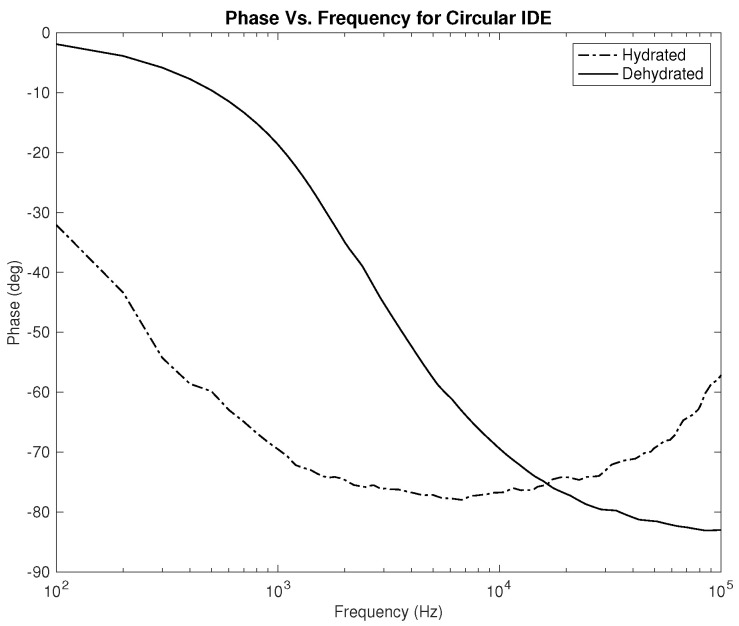
Simulation results—phase (Degree) of hydrated and dehydrated skin using the circular IDE design.

Three parameters were measured to be correlated with the hydration level—the resistance, capacitance, and phase. Plots showing the relationship between the applied frequency and the measured parameters are shown in Figure 6, Figure 7, Figure 8, Figure 9, Figure 10 and Figure 11. As it can be seen in Figure 6 and Figure 7, plotting the resistance for both hydrated and dehydrated skin shows clear distinguishable lines that represent both skin states in terms of amplitude. It is also noticed that the rectangular electrode exhibits higher resistance reading.

As for the capacitance shown in Figure 8 and Figure 9, the difference between both skin states is noticeable both in terms of the amplitude and slope, where they are much higher for the dehydrated skin. Unlike the resistance, the capacitance of the electrode is observed to give higher values for the circular-shaped electrode. However, if the capacitance is to be realized for body hydration classification, sweeping the frequency up to 1 kHz seems to be enough.

Lastly, the phase plots, which are identical for both designs as noticed in Figure 10 and Figure 11, exhibit a very interesting behavior where in some range of frequencies, the slope of both lines is opposite to one another, which can be used to classify hydration state. Nevertheless, for this parameter, in particular, the phase should be studied for several clinical tests on human subjects to examine the shift of the overlap between the two hydration states’ behavior depending on the physical variables of the subject.

### 2.3. Testing and Validation

To test and validate the BIA method, the rectangular IDE was fabricated in Hamad Bin Khalifa University labs using the Fuji Film Dimatix DMP-2850 inkjet printer. The electrode was fabricated using silver ink and Polydimethylsiloxane (PDMS) as a substrate. The validation was performed as per the testing procedure below, which has an IRB approval under IRB Protocol Number: QBRI-IRB 2021-09-109. The testing procedure was performed using two main devices: the BodyStat Multiscan 5000, which is a spectroscopy (BIS) device to measure the body composition and hydration, and the system designed in this paper, which was used to measure the bio-impedance of subjects. The electrode designed was connected to the E4990A impedance analyzer and strapped around the subjects’ wrists. In each test, the subject had their hydration measured using the BodyStat Multiscan as a reference, and then their bio-impedance was recorded using the designed system.

**Figure 12:**
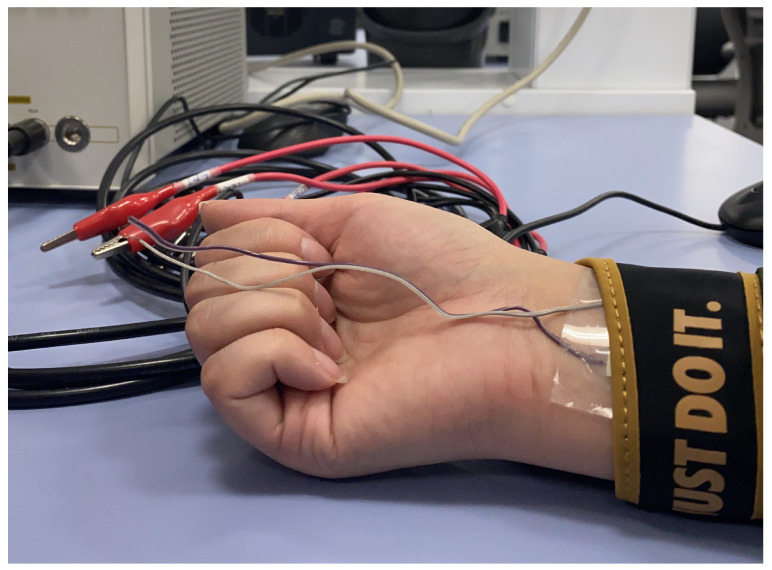
Testing procedure—the electrode strapped on the right wrist of the subject.

The following procedures were implemented for testing and validation:

1. Subjects were invited to undergo the tests 3 days a week for two weeks (a total of a maximum of 6 visits). Each visit was around 15–30 min long. The invitation included healthy subjects, male and female, aged between 18 to 60 years old. Pregnant women, cardiac patients, and patients on dialysis were excluded from the study.
2. In the first session, subjects’ information, including height, weight, and age, was collected to calibrate the BodyStat Multiscan measurements.
3. Subjects had their hydration level measured in all sessions using the BodyStat Multiscan, which was measured in percentage (%).
4. Subjects had their bio-impedance measured using the system designed in this project. The electrode was strapped around the subjects’ wrists. The subjects had the measurement taken once on the right wrist and once on the left wrist. Figure 12 shows the electrode strapped on the right wrist. Resistance and capacitance values were randomly measured in each measurement.

## 3. Results

In this paper, the results of testing on six different participants are presented. Four of the participants are male, two of whom are hydrated and two are dehydrated, two of the participants are female, one who is hydrated, and the other dehydrated. The hydration level and status of each participant were recorded using the BodyStat Multiscan device, which indicates males to be hydrated when their hydration level is between 55% to 65%, and females to be hydrated when their hydration level is between 50% to 60%. The hydration level of each participant is indicated in the legend of each figure.

**Figure 13:**
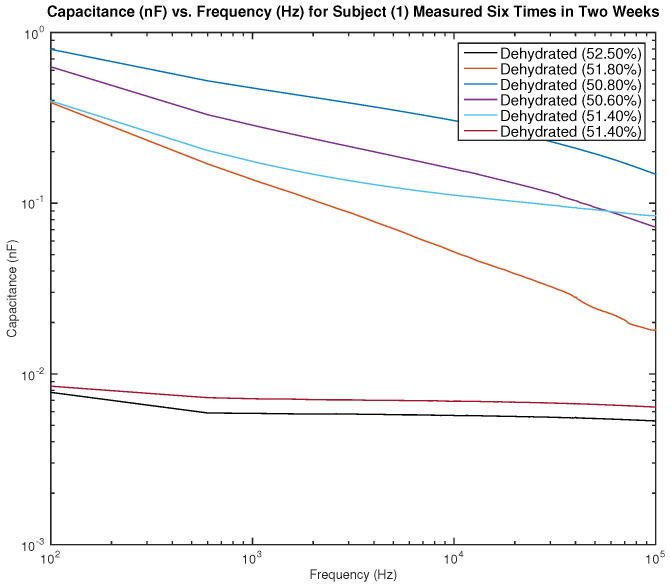
Testing results—capacitance (nF) vs. frequency (Hz) for subject 1 measured six times in two weeks on the left arm.

**Figure 14:**
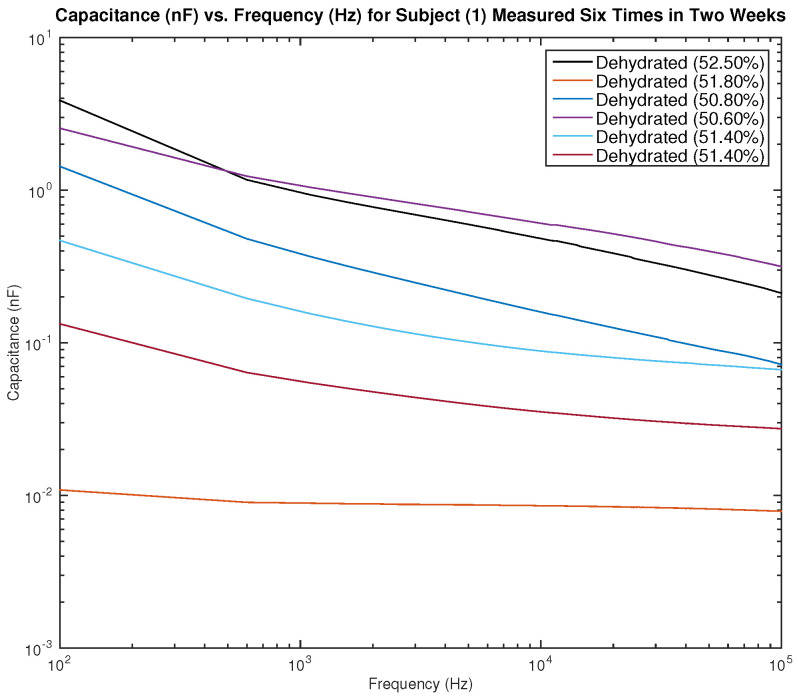
Testing results—capacitance (nF) vs. frequency (Hz) for subject 1 measured six times in two weeks on the right arm.

Figure 13 and Figure 14 show the measured capacitance for subject 1, a male, who is dehydrated (the average hydration level for a male is 55–65%). Figure 13 shows six different measurements taken over two weeks, all performed on the left arm, and Figure 14 shows six different measurements taken over two weeks, all performed on the right arm. It can be noticed in the figure that although the hydration levels are close and sometimes similar, the measured capacitance is not consistent.

**Figure 15:**
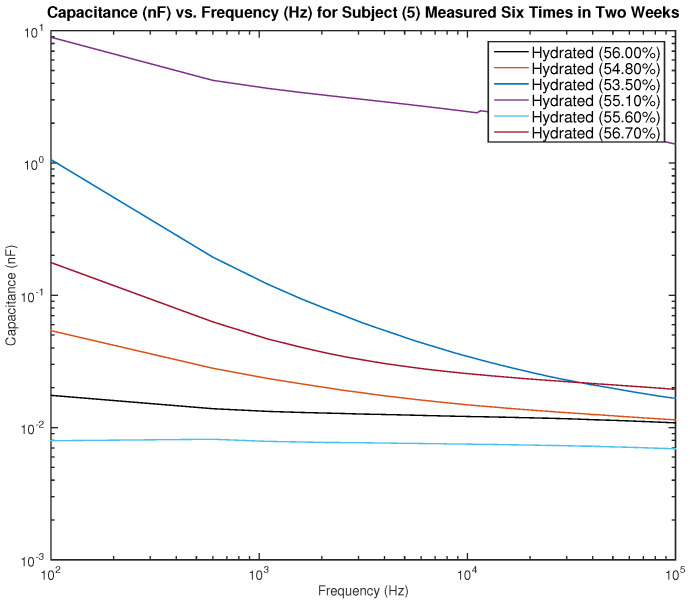
Testing results—capacitance (nF) vs. frequency (Hz) for subject 5 measured six times in two weeks on the left arm.

**Figure 16:**
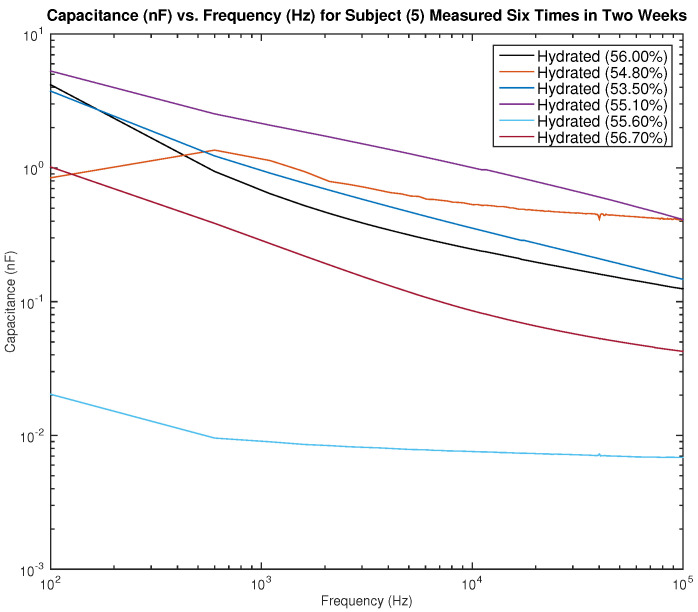
Testing results—capacitance (nF) vs. frequency (Hz) for subject 5 measured six times in two weeks on the right arm.

Figure 15 and Figure 16 show the measured capacitance for subject 5, a female, who is hydrated (the average hydration level for a female is 50–60%). Figure 15 shows six different measurements taken over two weeks, all performed on the left arm, and Figure 16 shows six different measurements taken over two weeks, all performed on the right arm. As noticed earlier with the results of subject 1, although the hydration levels are close and sometimes similar, the measured capacitance is not consistent.

**Figure 17:**
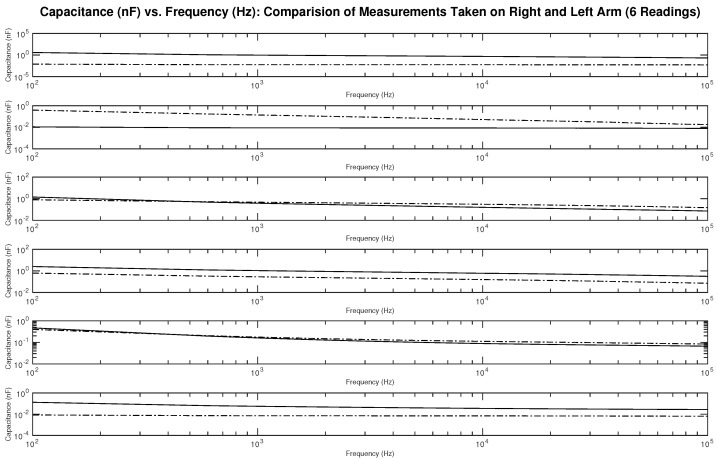
Testing results—capacitance (nF) vs. frequency (Hz): comparison of measurements taken on the right (solid line) and left (dashed line) arm.

Figure 17 shows the measured capacitance for subject 1, a male, who is dehydrated (the average hydration level for a male is 55–65%). The figure shows six different subplots presenting a comparison of the measurement taken on the right and left arm on the same testing day. It can be noticed from the figures some differences between the readings.

**Figure 18:**
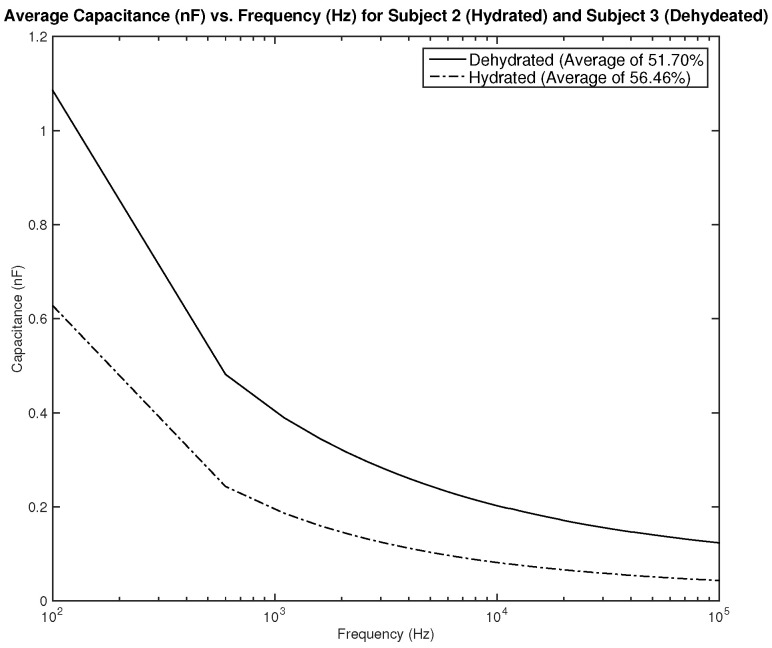
Testing results—capacitance (nF) vs. frequency (Hz): comparison between average measurement of hydrated and dehydrated subjects (Both Male).

**Figure 19:**
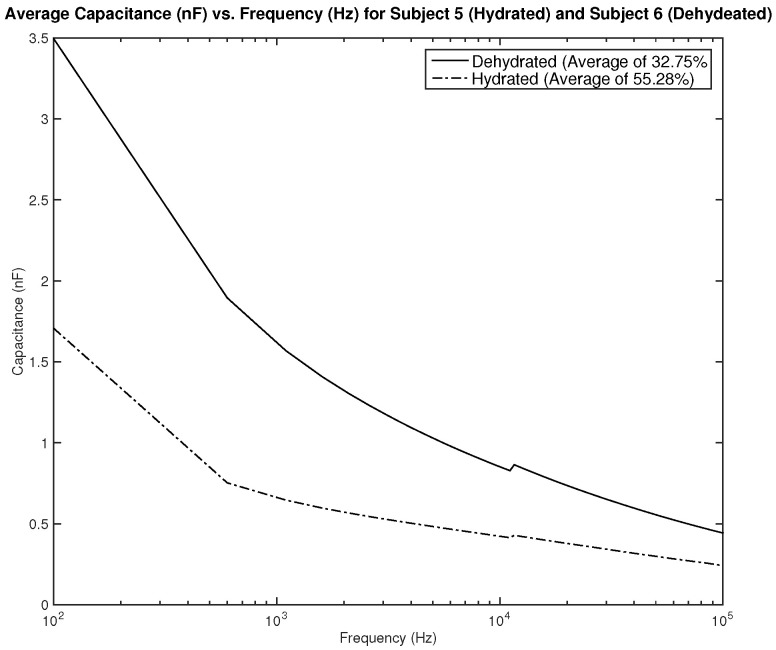
Testing results—capacitance (nF) vs. frequency (Hz): comparison between average measurement of hydrated and dehydrated subjects (Both Female).

Figure 18 and Figure 19 show the measured average capacitance of two subjects, one hydrated and one dehydrated. Figure 18 shows the measurements for subjects 2 and 3. Both subjects are male, subject 2 is hydrated with an average hydration level of 56.46%, and subject 3 is dehydrated with an average hydration level of 51.7%. As the results show, the difference in measured resistance is not significant, which can cause an issue in terms of the sensitivity of the sensor.

Figure 19 shows the measurements for subjects 5 and 6. Both subjects are female, subject 5 is hydrated with an average hydration level of 55.28%, and subject 6 is dehydrated with an average hydration level of 32.75%. The difference in measurements is significant and can be easily distinguishable. A dehydration classification can be concluded for a capacitance measurement of above 2 nF in frequencies between 100–1 kHz.

**Figure 20:**
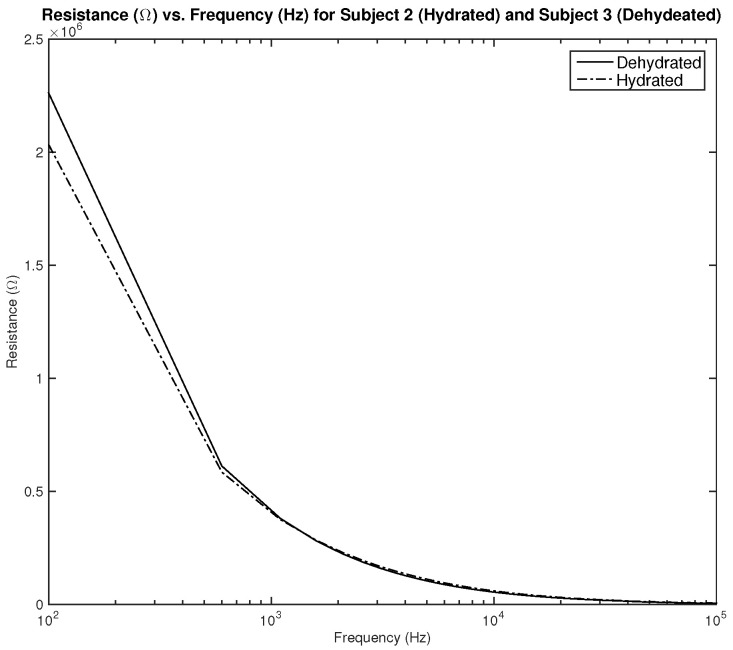
Testing results—resistance (Ω) vs. frequency (Hz) of hydrated and dehydrated subjects (both male).

Figure 20 shows the measured resistance for subjects 2 and 3. Both subjects are male, subject 2 is hydrated with an average hydration level of 56.46%, and subject 3 is dehydrated with an average hydration level of 51.7%. As the results show, the difference in measured resistance is not significant, which can cause an issue in terms of the sensitivity of the sensor.

**Figure 21:**
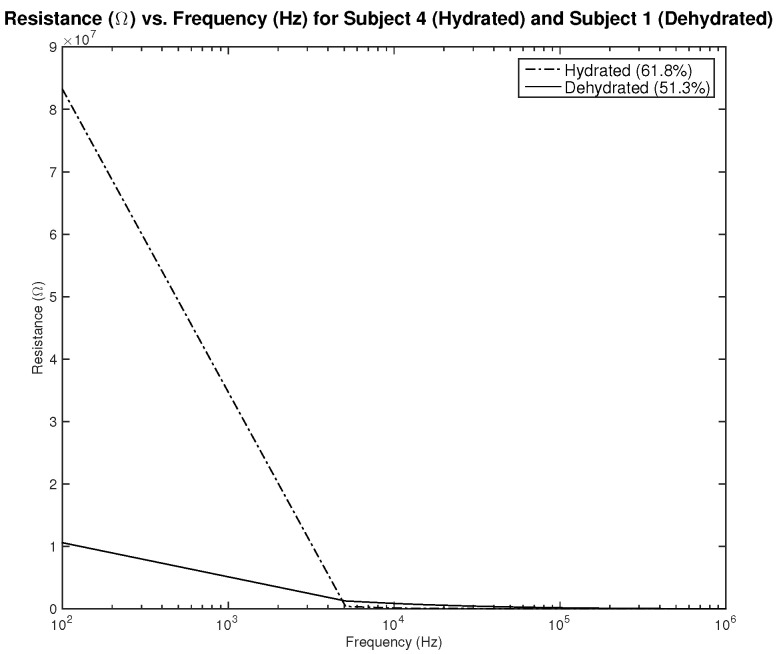
Testing results—resistance (Ω) vs. frequency (Hz) of hydrated and dehydrated subjects (both male).

**Figure 22:**
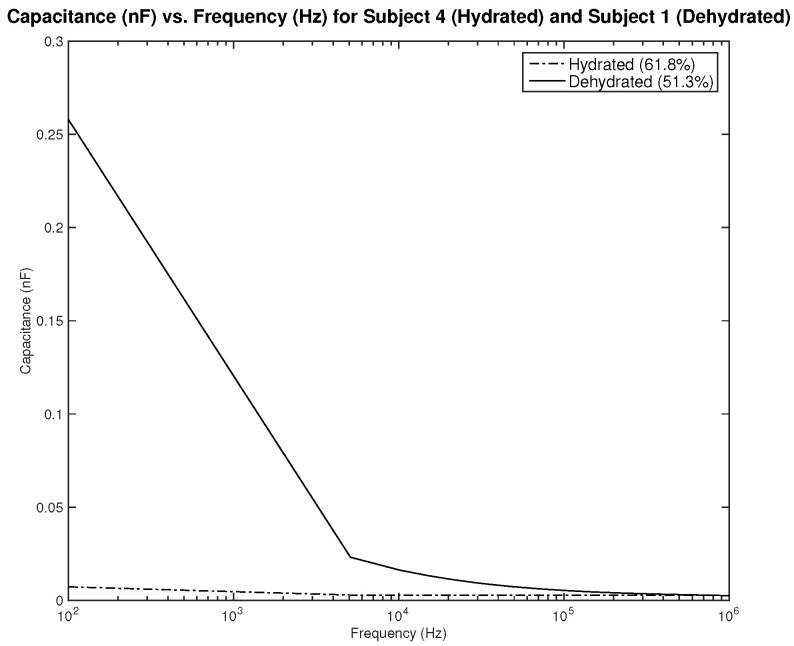
Testing results—capacitance (nF) vs. frequency (Hz) of hydrated and dehydrated subjects (both male).

**Figure 23:**
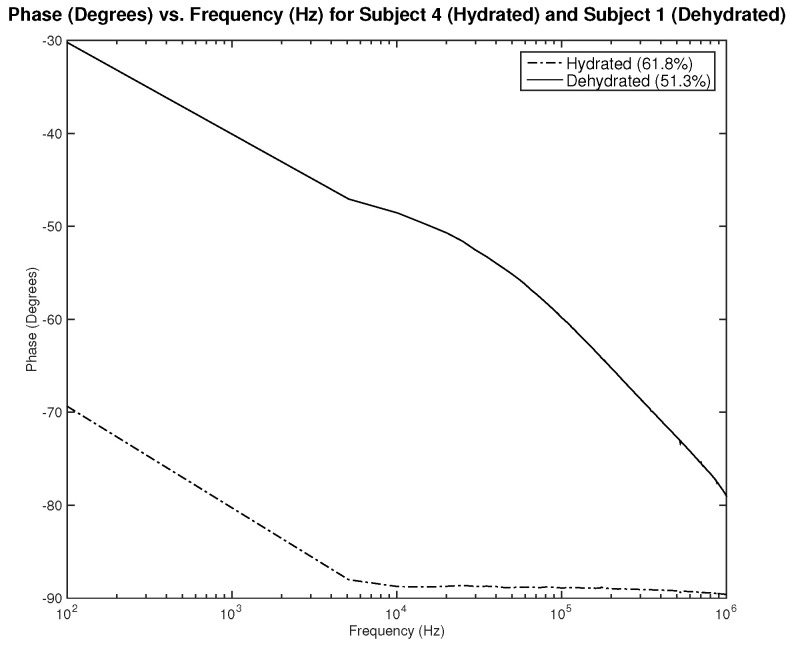
Testing results—phase (°) vs. frequency (Hz) of hydrated and dehydrated subjects (both male).

Figure 21, Figure 22 and Figure 23 show resistance, capacitance, and phase measurements of two male subjects, hydrated and dehydrated. The measurements were all taken at the same testing session. As noticed in the results, the difference in capacitance is minimal, while the difference in resistance and phase is distinguishable. This may indicate the need to assess the three parameters related to impedance to be able to classify hydration status.

## 4. Discussion

To design a dehydration sensor utilizing the BIA method, different parameters must be studied and optimized. This includes the shape and placement of the electrodes, the material used, the signal applied, and the measurement taken, which each affects the results and their interpretation. The simulation model presented in this paper provides a base model to study the design of the system, test the model, and validate it.

The first part of the study focuses on the simulation of the skin mode, the electrode design, and the measured parameters. The skin model takes into account the change in the dielectric properties of the skin based on the hydration status and the frequency of the applied signal. Two interdigitated electrode designs are chosen to be studied for this system. Both designs were simulated on top of the skin model in 3D mode. An alternating signal is applied to one electrode with a frequency ranging from 100 Hz to 0.1 MHz, while the other electrode is set to the ground. Three parameters, the resistance, capacitance, and phase, were measured and analyzed. As noticed, for both electrodes design, measurements for hydration and dehydration skin show the distinguishable difference that can be used to classify the hydration status of the skin. Higher resistance readings are recorded with the rectangular-shaped electrode, while the capacitance readings are higher in the circular-shaped electrode. The capacitance readings are noticed to be significant only in the low frequencies (up to 1 kHz). The phase measurements show different behavior compared to the resistance and capacitance, and changes in the slope can be noticed between low and high frequencies.

In the second part of the study, the BIA method is validated by testing it on two participants with two different hydration statuses. The participants were tested using the BodyStat 5000 to indicate their hydration status, and then their bio-impedance was measured using an electrode connected to an impedance analyzer. Most of the measurements acquired were of capacitance in three different levels of hydration states: severe dehydration, mild dehydration, and hydration. The initial results show a discrepancy between readings taken from the left arm compared to the right arm of the same subject in the same testing session. Discrepancies were also noted when the measurement was taken on the same subject in different testing sessions, even if there were no changes in hydration level. These may be caused by factors such as body temperature and body movement or environmental factors related to the testing settings. When comparing measurements taken on subjects, a significant difference in capacitance readings was noticed in the case of severe dehydration. However, the readings cannot help in classifying hydration status in the cases of mild dehydration. Assessing readings of all impedance values, resistance, capacitance, and phase, may be required to better identify accurate classification criteria. The findings above show the possibility of utilizing the BIA method in a hydration assessment without the need for tuning and calibrating with the subjects’ information, such as height, weight, and age. However, to be able to decide on the criteria on which hydration or dehydration is assessed, a large sample with a range of body water levels that varies from severe dehydration to hydration for both men and women is required. Hence, further clinical testing is needed to confidently set the classification criteria. Further investigation is also needed to study the reasons behind the changes in readings between the right and left arms and between different tests for the same subject. Further investigation can focus on reasons such as body temperature and body movement and the effect of both on hydration measurement.

## 5. Conclusions

Bio-impedance analysis has a great potential to be utilized as a noninvasive and cost-efficient method to assess hydration in wearable devices. While the concept of BIA is simple and easy to implement, the interpretation of the data and correlation of the results depends on several factors. In this paper, a simulation model combining the skin parameters and system design is presented as a base to study the dehydration monitoring system. The model studies the effect of electrode design and applied signal on the measured impedance, taking into account the changes in dielectric parameters of the skin based on hydration status. The designed rectangular IDE was printed, tested, and validated in the Hamad Bin Khalifa University labs. The preliminary results show that distinguishing between hydrated and dehydrated subjects can be accomplished by measuring the capacitance in the case of severe dehydration. However, the measurements are close and cannot be distinguished when the hydration and dehydration levels are close. The results have also shown discrepancies in readings between the right and left arm and between readings taken for the same subject on different days. This may indicate that some other factors are affecting the reading, such as body temperature and body movement. Clinical testing of the system will continue to improve the interpretation of the data, and the impedance analyzer design will be studied to fit the application of wearable devices.

## Figures and Tables

**Table 1:** Design parameters of rectangular and circular IDEs.

Parameter	Value	Unit (If Any)
Material	Sliver	
Number of Fingers	6	
Finger Length	1	mm
Spacing	1	mm
Collector Bar Width	1	mm
Contact Pads	4 × 4	mm × mm

## Data Availability

The data presented in this study are available on request from the corresponding author. The data are not publicly available as the study is still ongoing.
